# Inhibitory Effects of Dietary *N*-Glycans From Bovine Lactoferrin on Toll-Like Receptor 8; Comparing Efficacy With Chloroquine

**DOI:** 10.3389/fimmu.2020.00790

**Published:** 2020-05-12

**Authors:** Susana Figueroa-Lozano, Rivca L. Valk-Weeber, Renate Akkerman, Wayel Abdulahad, Sander S. van Leeuwen, Lubbert Dijkhuizen, Paul de Vos

**Affiliations:** ^1^Immunoendocrinology, Division of Medical Biology, Department of Pathology and Medical Biology, University Medical Center Groningen, University of Groningen, Groningen, Netherlands; ^2^Microbial Physiology, Groningen Biomolecular Sciences and Biotechnology Institute (GBB), Groningen, Netherlands

**Keywords:** *N*-glycosylation, bovine lactoferrin, inhibition, TLR-8, immunomodulation, autoimmune disorders

## Abstract

Toll-like receptor 8 (TLR-8) plays a role in the pathogenesis of autoimmune disorders and associated gastrointestinal symptoms that reduce quality of life of patients. Dietary interventions are becoming more accepted as mean to manage onset, progression, and treatment of a broad spectrum of inflammatory conditions. In this study, we assessed the impact of *N*-glycans derived from bovine lactoferrin (bLF) on the inhibition of TLR-8 activation. We investigated the effects of *N*-glycans in their native form, as well as in its partially demannosylated and partially desialylated form, on HEK293 cells expressing TLR-8, and in human monocyte-derived dendritic cells (MoDCs). We found that in HEK293 cells, *N*-glycans strongly inhibited the ssRNA40 induced TLR-8 activation but to a lesser extent the R848 induced TLR-8 activation. The impact was compared with a pharmaceutical agent, i.e., chloroquine (CQN), that is clinically applied to antagonize endosomal TLR- activation. Inhibitory effects of the *N*-glycans were not influenced by the partially demannosylated or partially desialylated *N*-glycans. As the difference in charge of the *N*-glycans did not influence the inhibition capacity of TLR-8, it is possible that the inhibition mediated by the *N*-glycans is a result of a direct interaction with the receptor rather than a result of pH changes in the endosome. The inhibition of TLR-8 in MoDCs resulted in a significant decrease of IL-6 when cells were treated with the unmodified (0.5-fold, *p* < 0.0001), partially demannosylated (0.3-fold, *p* < 0.0001) and partially desialylated (0.4-fold, *p* < 0.0001) *N*-glycans. Furthermore, the partially demannosylated and partially desialylated *N*-glycans showed stronger inhibition of IL-6 production compared with the native *N*-glycans. This provides evidence that glycan composition plays a role in the immunomodulatory activity of the isolated *N*-glycans from bLF on MoDCs. Compared to CQN, the *N*-glycans are specific inhibitors of TLR-8 activation and of IL-6 production in MoDCs. Our findings demonstrate that isolated *N*-glycans from bLF have attenuating effects on TLR-8 induced immune activation in HEK293 cells and human MoDCs. The inhibitory capacity of *N*-glycans isolated from bLF onTLR-8 activation may become a food-based strategy to manage autoimmune, infections or other inflammatory disorders.

## Introduction

Bovine lactoferrin (bLF) is a milk derived glycosylated protein with confirmed anti-viral (1), anti-bacterial, anti-fungal, anti-oxidant (2), and immunomodulating properties (3). It is present in bovine colostrum and mature milk at concentration of 1.5–5 mg/mL and 1.5–3 mg/mL, respectively (4). The human equivalent is present in at least ten times higher concentrations in breast milk (5). When mother milk is not an option or insufficiently produced, infants are receiving bLF via infant formula (3). Besides its use in infant formula, bLF is widely used as a functional food product and has many biopharmaceutical applications (6). Several studies have been dedicated to the characterization of the structure of bLF, mostly to the determination of its amino acid sequence (7) and *N*-glycan composition (8). However, the relationship between its structural make-up and biological functionality has been less explored. So far, most research has been dedicated to the activity against Gram-positive and Gram-negative bacteria of LF and the role of the protein core in such bactericidal properties (9, 10).

The impact of *N*-glycosylation of bLF, i.e., the attachment of glycan moieties to specific sites of the protein, on its biological activity has not been studied in much detail. Although the structural homology of bLF compared with human LF (hLF) is 77% (11), the glycosylation pattern of bLF, differs significantly from the human counterpart (7). While hLF has 3 *N-*glycosylation sites, bLF has 5 (12). The *N*-glycans of bLF consist of units of *N*-acetyl-galactosamine (GalNAc), *N*-acetyl-glucosamine (GlcNAc), galactose, mannose, fucose, and sialic acid [*N*-acetyl-neuraminic acid (Neu5Ac) and *N*-glycolyl-neuraminic acid (Neu5Gc)]. Neu5Gc is only present on bLF and not present on human LF (13). *N*-glycosylation determines many characteristics of the bLF, such as the conformation, folding, solubility, and resistance to protein proteolysis (14) and its iron-binding capacity (15). The heterogeneity in the glycan chain monosaccharide composition of bLF is considered to be relevant for the broad spectrum of biological effects of human and bLF (13).

Chromatographic analysis of whey proteins in bovine milk has shown pertinent differences in *N*-glycosylation composition between human and bLF (16). Human LF consists of 6% oligo-mannose type glycans and 52% of complex/hybrid type glycans, of which 48% of the glycans are sialylated and 88% fucosylated (16). In contrast, bLF consists of 65% oligo-mannose and 4% complex/hybrid type glycans. Of these glycans 31% is sialylated and 9% fucosylated (16). The glycosylation pattern of bLF is known to change during lactation as a mechanism to respond to a broader spectrum of pathogens (17). Most of these studies were performed with mixtures of bLF and not with the *N*-glycans alone as it is cumbersome to obtain large enough samples to perform functional studies. New analytical techniques have changed this (18) and led to the characterization of structural components responsible for the anti-oxidant, iron binding (19) and immunomodulatory capacity of bLF (20).

The immunomodulatory capacity is another important biological activity of bLF (21). The oral administration of bLF has been shown to attenuate both, human and non-human immune inflammatory responses (22). This modulation is attributed to the ability of bLF to bind to lipopolysaccharides (LPS) and activate TLR-4 (23). LF has also been reported to enhance the activation of T-cells through modulation of dendritic cell responses (24). Most recently, it has been shown by us that the glycosylation pattern of bLF determines the TLR modulating capacity of bLF. A property of bLF *N*-glycans is that they antagonize the activation of TLR-8 (20).

Toll-like receptor 8 recognizes structural patterns of viral and bacterial ssRNA and it is expressed in lymphocytes, monocytes (Mo), and intestinal epithelial cells (IEC) (25). Additionally, TLR-8 can recognize self-RNA (26) and a small synthetic antiviral imidazoquinoline called resiquimod (R848). The dysregulation or overexpression of TLR-8 has been found to contribute to the pathogenesis and progression of autoimmune disorders, such as rheumatoid arthritis (27), systemic sclerosis (28), and inflammatory bowel disease (29). Inhibition of endosomal TLRs, such as TLR-8 has great therapeutic potential for the treatment of autoimmune diseases (30–32) as shown for the TLR7/8/9 antagonist chloroquine (CQN). Therefore, the inhibition of TLR-8 activation might be instrumental for the management of many inflammatory conditions (32, 33).

In experimental settings, the TLR-8 activation by RNA is mimicked with compounds such as HIV-derived ssRNA40 and R848. Although both compounds activate TLR-8 via the adaptor molecule Myd88, it has been shown that CQN inhibits ssRNA40 induced activation of TLR-8 but only to a minor extent antagonizes R848 induced TLR-8 activation (34). These differences in efficacy of TLR-8 inhibition by CQN have been attributed to different mechanisms of inhibition (35). Endosomal TLRs require the acidification of the endosome for their activation. By preventing this process, CQN inhibits TLR-8 signaling (36). The other mechanism involves two possibilities: either the direct interaction of CQN with TLRs ligands or the disruption of the configuration changes of the TLR-8 dimer structure required for activation (37).

In order to gain more insight in the interactions of the isolated *N*-glycans from bLF and its partially demannosylated and partially desialylated counterparts, we compared the inhibitory effect of *N*-glycans on the ssRNA40 and R848 induced activation of TLR-8 in HEK293 cells. In addition, we investigated whether these *N*-glycans were able to exert different inhibitory effects on the activation of TLR-8 and the production of cytokines in MoDCs.

## Materials and Methods

### Preparation of *N*-Glycan Fractions

Bovine lactoferrin was provided by FrieslandCampina Domo (Amersfoort, Netherlands). PNGase F (*Flavobacterium meningosepticum*) was from New England Biolabs (Ipswich, United Kingdom). Bio-Beads SM-2 were purchased from Bio-Rad Laboratories (Veenendaal, Netherlands). Solid phase extraction (SPE) of the large-scale PNGase F digests was performed on, prepacked C18 (CEC18, 200 mg;3mL, Screening Devices, Amersfoort, Netherlands) and graphitized carbon SPE cartridges (Extract-clean carbograph, 150 mg;4mL, Grace, Columbia, United States). In order to alter the content of mannose and sialic acid on the glycan chains of bLF, the native protein was incubated with α-mannosidase and sialidase as reported previously (20).

**FIGURE 1:**
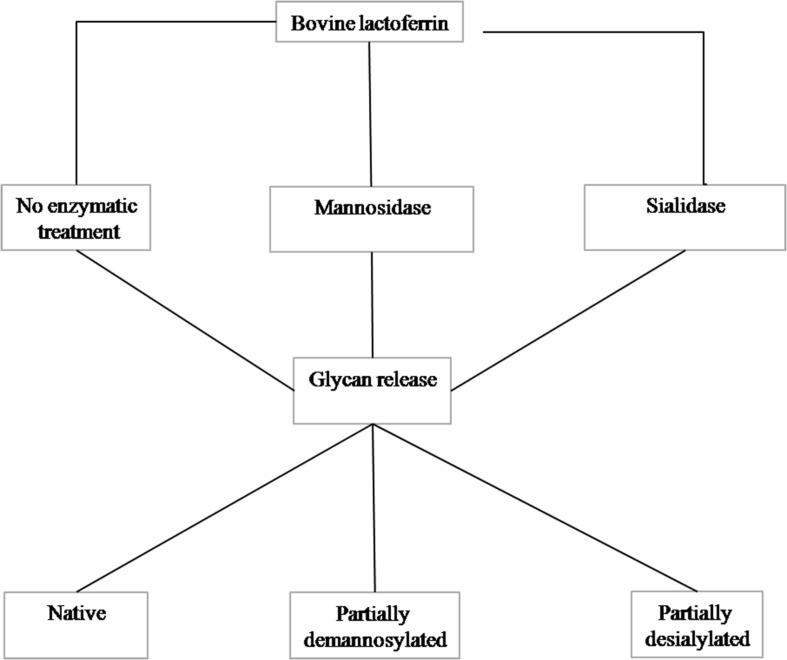
Schematic representation of the preparation of the *N*-glycan fractions.

Purified glycans were obtained using the method described by Valk-Weeber et al. (18). One gram of bLF was dissolved at a concentration of 7.5 mg/mL in 100 mM sodium phosphate buffer (pH 7.5). SDS was added at a 1:1 w/w protein: SDS ratio and β-mercaptoethanol (Sigma) was added to a concentration of 1% v/v. The protein was denatured by heating at 85°C for 30 min. Denatured protein was alkylated by addition of iodoacetamide (Sigma) to a concentration of 20 mM (55°C; 30 min). This step was performed in the dark because of instability of iodoacetamide in light. Nonidet P-40 substitute (NP-40, Sigma) was added at a final concentration of 1% v/v. PNGase F was added at a concentration of 50 U/mg glycoprotein and the solution incubated overnight at 37°C with continuous agitation. Detergents were removed with Bio-Beads SM-2 (Bio-Rad) using 1 g of beads: 10 mg of digested protein. Protein was removed by 30 kDa centrifugal MWCO filters (Amicon Ultra, Merck Millipore, Tullagreen, Cork, Ireland). The partially purified digest was further purified by C18 and graphitized carbon SPE. Aqueous glycan samples were loaded onto conditioned C18 material and the flow through, containing glycans, was collected and loaded onto the graphitized carbon. The graphitized carbon was washed with MilliQ water to remove salts and finally the glycans were eluted with 25% acetonitrile containing 0.1% TFA. Elution fractions were neutralized with 2% ammonia, the acetonitrile evaporated under N_2_ and lyophilized. Residual detergent traces were removed by washing the lyophilized *N*-glycans with 5 mL 100% ice cold (−20°C) acetone. Purity of the resulting glycan products was determined by monosaccharide analysis and 1D ^1^H NMR spectroscopy as described (18). A schematic overview of the enzymatic treatment is presented in Figure 1.

### Cell Culture of HEK293-TLR-8 Cell Line

HEK-Blue^TM^ hTLR-8 reporter cell lines and reagents, such as selection media Quanti-Blue^TM^ reagent, and the agonists single-stranded GU-rich oligonucleotide complexed with LyoVec (ssRNA40/LyoVec^TM^) and R848 (resiquimod), were purchased from InvivoGen (Toulouse, France). This cell line carries a construct for SEAP coupled to the NF-κB/AP-1 promoter. The HEK-Blue^TM^-hTLR8 cell line was maintained in DMEM (Life Technologies Europe B.V) containing 10% heat inactivated fetal bovine serum (FBS), L-glutamine (2.0 mM-Sigma-Aldrich Chemie B.V), glucose (4.5g/L Sigma-Aldrich Chemie B.V), penicillin-streptomycin (50 U/mL–50 μg/mL Sigma-Aldrich Chemie B.V), and normocyn (100 μg/mL Sigma-Aldrich Chemie B.V). HEK-Blue^TM^ hTLR8 cells were grown to approximately 80% of confluence. After culturing for 3 passages, all reporter cell lines were maintained on selection media according to the manufacturer’s protocol.

### TLR Activation and Inhibition Assays in Reporter Cell Line Experiments

HEK-Blue^TM^ hTLR8 cell lines were detached from the bottom flask after which the cells were centrifuged and re-suspended according to manufacturer’s protocol. Later the cells were seeded at 2.2 × 10^5^ cells/mL in 96 well plates at 100 μL per well. Inhibition of TLR-8 activation was studied by comparing the NF-κB release of TLRs agonists (ssRNA40, R848) with the NF-κB release of cells co-incubated with TLR agonist and inhibitors. TLR-8 inhibitor, CQN, was acquired from Sigma-Aldrich Chemie B.V. The native, partially demannosylated and partially desialylated *N*-glycans were prepared as reported previously (20). The cells were incubated for 1 h with 10 μL of inhibitor (CQN or *N*-glycans fractions); afterward 10 μL of agonist was added. All compounds were dissolved in culture medium. The plates were incubated for 24 h at 37°C and 5% CO_2_. After this period, 20 μL of supernatant were mixed with 180 μL of Quanti-Blue^TM^ in flat bottom 96 well plates. The plate was incubated for 1 h at 37°C and 5% CO_2_. After incubation, the activity of SEAP turns Quanti-Blue^TM^ substrate to blue color. The NF-kB release was quantified at 650 nm in a Benchmark Plus Microplate Reader using Microplate Manager 5.2.1 version for data acquisition. The assays were performed with 4 technical repeats and each experiment was at least repeated 4 times.

### Monocyte Magnetic Isolation and Human Monocyte-Derived Dendritic Cells (MoDCs) Differentiation

#### Ethics Statement

Blood sampling of human volunteers was conducted within the University Medical Center Groningen (UMCG), Netherlands. Written informed consent was obtained and data was analyzed and presented anonymously. This research and consent procedure have been approved by the Ethical Review Board of the UMCG, as documented in the approved application “2007/255.”

#### Human Peripheral Blood Mononuclear Cells (PBMCs) Isolation and Monocyte (Mo) Purification

Human PBMCS were obtained using density gradient centrifugation. Whole blood was collected in EDTA tubes. The blood was diluted 1:1 with PBS free of calcium and magnesium. PBMCs were obtained by a Ficoll density gradient isolation (Lymphoprep, Stemcell Technologies, Cologne, Germany). From the freshly isolated PBMCs, CD14^+^ Mo were isolated using the EasySep Human Monocyte Isolation Kit (negative selection kit, Stemcell Technologies, Cologne, Germany) according to manufacturer’s instructions.

### MoDCs Differentiation and Maturation

Purified Mo were seeded in IMDM medium supplemented with 1% FBS and 1000 ng/mL of granulocyte-macrophage colony stimulating factor (GM-CSF) and 500U/mL of interleukin-4 (IL-4) (ImmunoTools, Germany) for a total of 6 days in 24-well culture plates at a density of 5 × 10^5^ cells/mL (38).

### Inhibition of TLR-8 Activation in MoDCs Experiment

Monocyte-derived dendritic cells were cultured in 96-well plates at a density of 50,000 cells per well. Non-treated cells served as negative control. To inhibit TLR-8 activation, CQN was used at a concentration of 10 μg/mL. The native, partially demannosylated and partially desialylated *N*-glycans isolated from bLF were tested to examine their ability to inhibit TLR-8 activation. Cells were either incubated for 1 h with CQN or with the *N*-glycan samples. After this time, cells were co-incubated with the agonist ssRNA40 for 24 h. The supernatant was collected for further analysis.

### Real-Time PCR Measurement of TLR8 mRNA Expression

Total RNA was isolated and purified from Mo and MoDCs with TRIzol reagent (Invitrogen). Superscript II reverse transcription kit (Invitrogen) was used. The levels of TLR-8 isoform Hs00607866_mH probe (Thermo Fisher Scientific) were measured using real-time PCR Taqman assay. Samples were run in triplicate. The relative expression of the TLR-8 isoform was normalized to the housekeeper GAPDH and calculated with the 2^−ΔΔCt^ method (39).

### Cytokine Detection by Enzyme-Linked Immunosorbent Assay (ELISA)

The culture supernatants of the MoDCs were removed after 24 h. The production of IL-6, IL-10 and TNF-α was measured in 96-well microtiter plates according to the manufacturer’s instructions (R&D Systems, DuoSet). Standard curves and sample concentrations were calculated based on the mean of triplicates for each sample.

### Statistical Analysis

Statistical analysis was performed using Graphpad 7. Normal distribution of the data sets was tested using Shapiro-Wilk normality test. Data was normally distributed and expressed as mean ± SEM. Statistical comparisons were performed using one-way ANOVA followed by *post hoc* Tukey’s multiple comparison test to show individual differences. A *p*-value < 0.05 was considered significantly different. Statistical differences in the release of NF-κB in the HEK293 cells expressing hTLR-8 were evaluated using paired *t*-test. A *p*-value < 0.05 was considered significantly different.

## Results

### Structural Characterization of Isolated Glycans From bLF

To obtain *N*-glycans from bLF in milligram amounts, which is needed to perform functional studies, a large-scale protocol for the purification and characterization of isolated *N*-glycans from bLF was developed (18). Prior to glycan release, the *N*-glycans were enzymatically treated to reduce the content of mannose and sialic acid. These modifications were done to study the impact of such reductions on the inhibition of TLR-8 activation in reporter cell lines (HEK293 hTLR-8) and in MoDCs.

**FIGURE 2:**
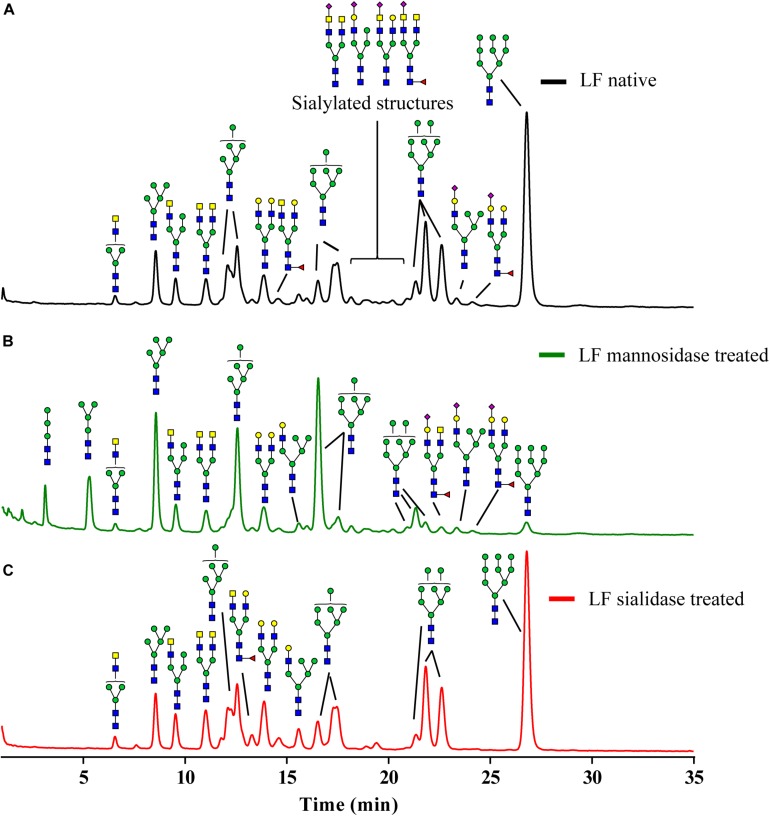
Glycoprofile of the *N*-glycans isolated from bLF. **(A)** Native isolated *N*-glycans, **(B)** partially demannosylated *N*-glycans, and **(C)** partially desialylated *N*-glycans.

As indicated in Figure 2A, the glycan composition of native *N*-glycans on bLF consists mainly of oligomannoses Man-5 to Man-9 and a small number of sialylated biantennary glycans. The mannosidase treatment (Figure 2B), reduced the mannose content with 11%, based on the monosaccharide analysis (data not shown). However, this overall reduction is not the only change induced by the mannosidase treatment, as the pattern of the oligomannose glycans was drastically altered. While Man-3 to Man-7 structures were still present, Man-8 and Man-9 had virtually disappeared. The characterization of the *N*-glycans treated with sialidase is presented in Figure 1C. Based on the monosaccharide analysis, sialic acid content was reduced by 94%. This is also reflected by the absence of sialylated structures and the increase of desialylated variants (Figure 2C).

### Differential TLR-8 Inhibition by *N*-Glycans Isolated From bLF

We have previously found that TLR-8 is inhibited by the *N*-glycans isolated from bLF (20). Inhibition of endosomal TLR-8 signaling is a possible approach to modulate the immune response in autoimmune diseases (30–32) as shown for CQN. If isolated *N*-glycans from bLF exert similar effects as TLR-8 antagonists, they may provide a food-based strategy for the management of inflammatory disorders and their intestinal symptoms.

It is unknown whether the *N*-glycans with different monosaccharide compositions isolated from bLF have direct interaction with the receptor ligands or directly with the TLR-8 dimer. Here, we investigated whether the TLR-8 inhibitory effects of *N*-glycans, either with or without reduction in mannose and sialic acid content, are dependent on the agonist used for stimulation and whether the inhibition is dose dependent. To this end, HEK293 cells expressing hTLR-8 were pre-exposed for 1 h to different concentrations of either CQN or the *N*-glycans and subsequently exposed to agonists ssRNA40 and R848.

**FIGURE 3:**
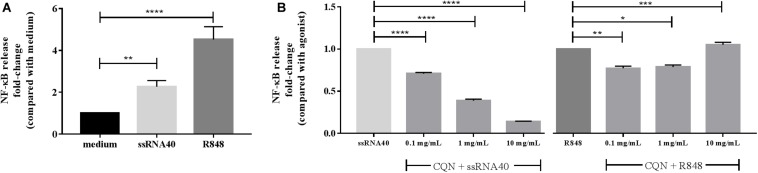
Inhibitory effects of CQN on ssRNA 40 and R848 induced activation of TLR-8. The TLR-8 agonists ssRNA40 (50 μg/mL) and R848 (100 μg/mL) were used to stimulate TLR-8 expressed in HEK293 cells after pre-incubation with chloroquine (CQN) for 1 h at 0.1, 1, and 10 mg/mL (*n* = 4). **(A)** Fold-change activation induced by CQN compared with the untreated cells/medium. **(B)** Fold-changes in the inhibition of ssRNA40 or R848 induced TLR-8 activation. NF-κB release was measured by spectrophotometry at 650 nm. Data is represented as mean ± SEM. Statistical differences were measured using One-way ANOVA and *post hoc* Tukey’s test. Significant differences compared to ssRNA40 and R848 are indicated by *p < 0.05, **p < 0.01, ***p < 0.001, and ****p < 0.0001.

We first determined the TLR-8 stimulating capacity of ssRNA40 and R848 in HEK293 cells expressing hTLR-8. As shown in Figure 3A, ssRNA40-induced TLR-8 activation was 2.3-fold (*p* < 0.001) higher compared to the medium control while R848 induced TLR-8 activation 4.5-fold (*p* < 0.0001). This activation was differently suppressed by CQN. CQN inhibited (*p* < 0.0001) as previously reported, ssRNA40 induced TLR-8 activation more efficiently than TLR-8 activation by R848. The inhibition of ssRNA40 induced TLR-8 activation was CQN dose dependent (40). CQN at concentrations of 0.1, 1, or 10 mg/mL inhibited TLR-8 induced activation 0.7 (*p* < 0.0001), 0.5 (*p* < 0.001), and 0.2-fold (*p* < 0.0001), respectively.

The inhibition of TLR-8 by CQN was less strong when the agonist used for stimulation was R848. A statistically significant but mild inhibition occurred at 0.1 mg/mL (*p* < 0.01) and at 1 mg/mL (*p* < 0.05) but at a concentration of 10 mg/mL CQN significantly co-stimulated the R848 induced TLR-8 activation (*p* < 0.001) (Figure 3B).

**FIGURE 4:**
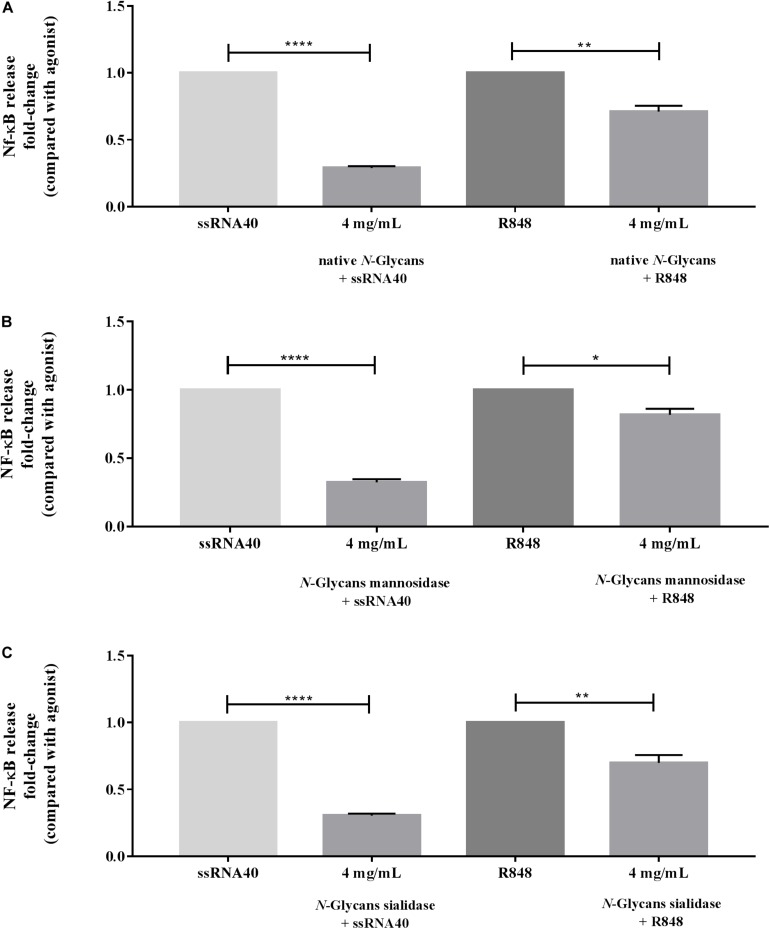
Impact of the native, partially demannosylated and partially desialylated *N*-glycans on the inhibition of TLR-8 activation induced with ssRNA40 or R848. HEK293 cells expressing hTLR-8 were first incubated with **(A)** native *N*-glycans, **(B)** *N*-glycans treated with mannosidase, and **(C)** *N*-glycans treated with sialidase at 4 mg/mL (*n* = 5). After 1 h, the TLR-8 agonists ssRNA40 (50 μg/mL) and R848 (100 μg/mL) were used to stimulate the cells. NF-κB activation was measured by spectrophotometry at 650 nm. Data is represented as mean ± SEM. Statistical differences were measured using paired *t*-test. Significant differences compared to ssRNA40 and R848 are indicated by *p < 0.05, **p < 0.01, and ****p < 0.0001.

Figure 4 demonstrates the inhibitory capacity of the different *N*-glycans from bLF on TLR-8 induced activation by ssRNA40 and R848. The HEK293 cells were incubated with native *N*-glycans, *N*-glycans treated with mannosidase, and *N*-glycans treated with sialidase at 1, 2, and 4 mg/mL. No dose-response relationship was found. The native *N*-glycans highly reduced ssRNA40 induced TLR-8 activation by 0.43-fold (*p* < 0.0001). This inhibitory effect was already strong at a concentration of 1 mg/mL and was not further increased at the higher dose (4 mg/mL) Figure 4A. In contrast, when R848 was used to stimulate the cells, only the high, 4 mg/mL, concentration of the native *N*-glycans induced a significant inhibition of the R848-induced TLR-8 activation and this inhibition was still minor (Figure 4A).

As shown in Figure 4B, the partially demannosylated *N*-glycans inhibited the ssRNA40 induced TLR-8 activation significantly at all tested concentrations and to a similar extent as native *N*-glycans. Although the partially demannosylated *N*-glycans slightly reduced the R848 induced TLR-8 activation, the inhibition was less strong at 4 mg/mL (*p* < 0.05).

The partially desialylated *N*-glycans inhibited ssRNA40 induced TLR-8 activation significantly (*p* < 0.0001) at all tested concentrations and also to a similar extent as native *N*-glycans. For R848 induced TLR-8 activation again a slight inhibition was observed at an *N*-glycan concentration of 1 mg/mL which reached significance at 2 and 4 mg/mL (*p* < 0.01 and *p* < 0.001, respectively) (Figure 4C).

### Inhibitory Effects of bLF *N*-Glycans in MoDCs

Next, we determined the ability of the *N*-glycans to modulate TLR-8 induced activation of human MoDCs. As inhibitory effects of CQN and of the different isolated *N*-glycans from bLF were strong on ssRNA40 induced TLR-8 activation and only minor on activation induced by R848, we only tested inhibition of ssRNA40 induced TLR-8 activation of MoDCs. We confirmed expression of TLR-8 in the donor cells (Supplementary Figure S1). The secretion of IL-6, IL-10, and TNF-α was quantified in samples after 24 h.

As sensitivity of the HEK293 and MoDCs for ssRNA40 induced TLR-8 was found to be different, we first performed a dose-response study with ssRNA40 and CQN induced IL-6 secretion in MoDCs (Supplementary Figure S2). Based on the results we selected the following concentrations: MoDCs were incubated for 1 h with 10 μg/mL CQN and 25 μg/mL of the *N*-glycans. Afterward, cells were stimulated with 10 μg/mL ssRNA40.

**FIGURE 5:**
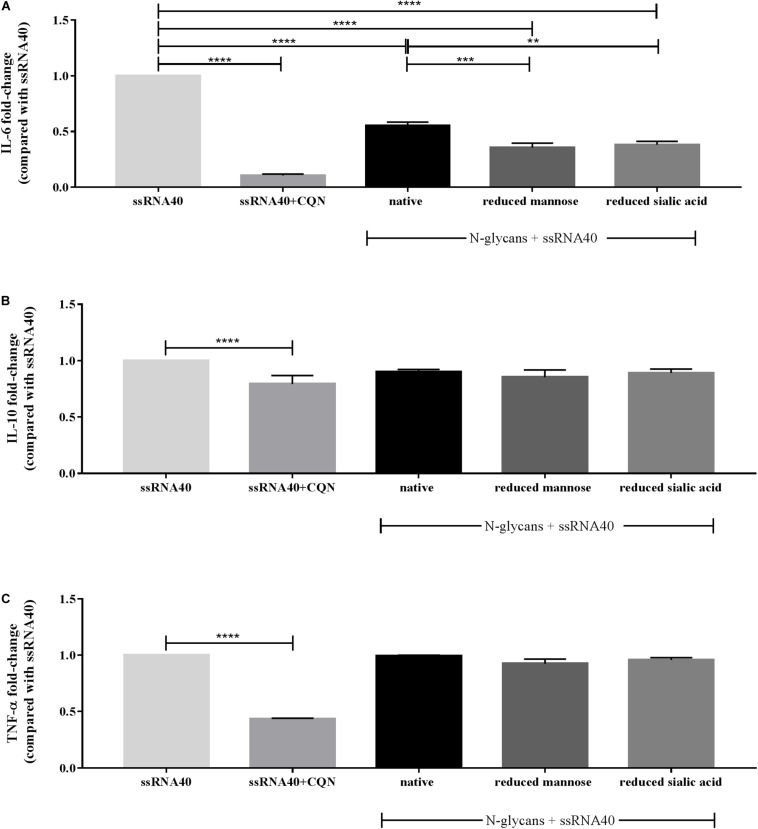
Effect of CQN and isolated native and modified *N*-glycans on the secretion of IL-6, IL-10, and TNF-α by MoDCs. TLR-8 agonist ssRNA40 (10 μg/mL) was used to stimulate the cells after pre-incubation with CQN (10 μg/mL) and the native and modified *N*-glycans at 25 μg/mL. The cytokine secretion of **(A)** IL-6, **(B)** IL-10, and **(C)** TNF-α is expressed in fold-change compared to ssRNA40 induced secretion of the cytokines. Data is expressed as mean ± SEM. Data represent 4 PBMCs isolations. Statistical differences were measured using One-way ANOVA and *post hoc* Tukey’s test. Significant differences compared to ssRNA40 are indicated by **p < 0.01, ***p < 0.001, and ****p < 0.0001.

As shown in Figure 5A, CQN at a concentration of 10 μg/mL very strongly suppressed ssRNA40 induced IL-6 secretion. This was less with the *N*-glycans which were tested at a higher concentration but the difference in IL-6 secretion was still significant. The native *N*-glycans inhibited the ssRNA40 induced IL-6 secretion 0.5-fold (*p* < 00001). The secretion of IL-6 was decreased 0.3-fold and 0.4-fold (*p* < 0.0001), by the partially demannosylated and desialylated *N*-glycans, respectively. Furthermore, the secretion of IL-6 for both partially demannosylated (*p* < 0.001) and partially desialylated (*p* < 0.01) *N-*glycans was significantly stronger than that of native *N*-glycans.

The secretion of the regulatory cytokine IL-10 was mildly affected by CQN and unaffected by the native, partially demannosylated, and partially desialylated *N*-glycans (Figure 5B). The ssRNA40 induced TNF-α secretion was strongly inhibited by CQN but was not reduced by the treatment with the *N*-glycans (Figure 5C).

## Discussion

In this study, we determined and compared the inhibitory effects of *N*-glycans isolated from bLF on the activation of TLR-8. Also, we assessed its immunomodulatory effects in human dendritic cells. The impact was compared with a pharmaceutical agent, i.e., CQN, that is clinically used to antagonize endosomal TLR activation (27). Previously, we have reported that dietary *N*-glycans isolated from bLF are inhibitors of ssRNA40 induced TLR-8 activation in reporter cell lines (20). Endosomal activation of TLRs is critical for host defense. However, excessive stimulation has been linked with the development of autoimmune disorders. TLR-8 specifically plays a role in autoimmune disorders because it is involved in the regulation of TLR-7 and TLR-9 signaling and a direct link has been found between the dysregulation of TLR-8 activation and pathological inflammation (25, 41).

Toll-like receptor 8 unlike other TLRs exists in inactive dimeric form before ligand recognition. Its activation is a multistep process (42). The activation of TLR-8 requires an acidification step, which occurs in the endosome (43). It is followed by the binding of the TLR-8 inactive dimer to ssRNA40 or R848. The dimerization interface of TLR-8 undergoes structural changes that enable ligand recognition, dimer activation, and downstream signaling (44). Compounds like CQN, which is a weak base, accumulate in this endosomal compartment and as a result it suppresses the activation of TLR-8 (45). The *N*-glycans are small molecules and they are mostly non-charged. Only the native and partially demannosylated *N-*glycans may carry a negative charge due to the presence of sialic acid but as they did not have a different degree of inhibition as the native *N-*glycans it is unlikely that the *N-*glycans inhibit TLR-8 activation by altering the endosomal acidic environment.

Endosomal acidification is not the only mechanism of inhibition identified for antagonists (33). TLR-8 inhibition can occur by at least two possible other ways. Antagonists can sequestrate the ligand for TLR-8 or it can hinder the structural reorganization of the receptor (44). It has been reported that the inhibitory capacity of CQN depends on the type of TLR stimuli used (40). As observed in this study, CQN antagonizes the release of NF-κB when ssRNA40 induced the activation of TLR-8. CQN at low concentrations had a mild inhibitory effect and at high concentration manifested a co-stimulatory effect. In the case of CQN, it has been found that it interacts with ssRNA40, making it unavailable for binding with TLR-8. There are no indications that the *N*-glycans induce inhibition of TLR-8 by binding ssRNA40.

Obstruction of the structural reorganization of the TLR-8 dimer has been shown as an inhibition mechanism by Zhang et al. (35). The inhibition of R848 induced activation of TLR-8 occurs at the antagonist binding pocket, where antagonists bind to prevent conformational changes required for activation (35). The *N*-glycans strongly inhibited the ssRNA40 induced TLR-8 activation, but exerted milder antagonistic effects on the R848 induced TLR-8 activation. This may suggest that the antagonistic effect of *N*-glycans is due to the direct interaction of the *N*-glycans with the TLR-8 dimer.

**FIGURE 6:**
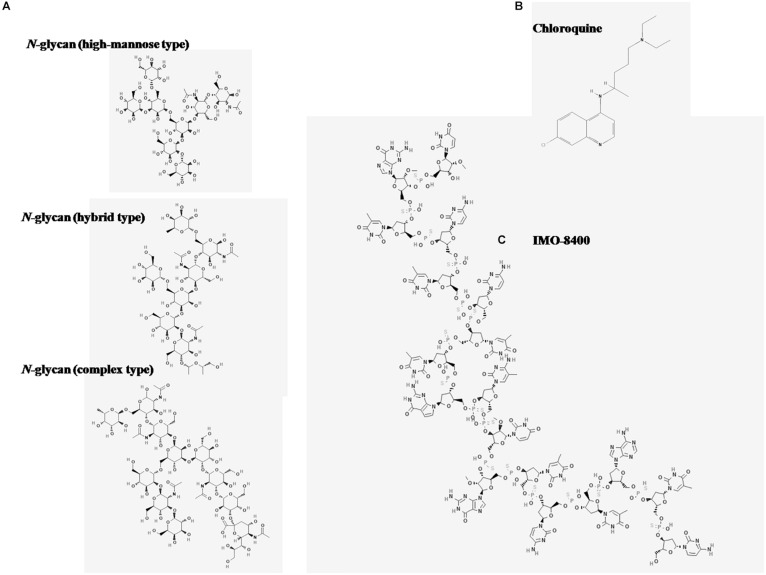
Chemical structures of antagonists of TLR-8 activation. Images were obtained from the National Center for Biotechnology Information. **(A)** Three major N-glycan types are depicted: High mannose type, PubChem Database. CID = 91852027, https://pubchem.ncbi.nlm.nih.gov/compound/91852027; hybrid type, PubChem Database. CID = 91860397, https://pubchem.ncbi.nlm.nih.gov/compound/91860397; complex type, PubChem Database. CID = 71297682, https://pubchem.ncbi.nlm.nih.gov/compound/71297682. **(B)** Chloroquine (CQN), PubChem Database. Chloroquine, CID = 2719, https://pubchem.ncbi.nlm. nih.gov/compound/Chloroquine. **(C)** IMO-8400 PubChem Database. Bazlitoran, CID = 119058029, https://pubchem.ncbi.nlm.nih.gov/compound/Bazlitoran.

The inhibitory capacity of the *N*-glycans may be linked to their structural characteristics. While TLR-8 recognizes nucleic acid sequences and structures containing tricyclic heterocycles, most endosomal TLRs antagonists, such as CQN, contain phenyl substituted bicyclic heterocyclic rings (33). Recently a new class of compounds called immune modulatory oligonucleotides (IMO), have shown inhibitory activity on endosomal TLRs (46). A structural similarity between the IMO compounds and the *N*-glycans is that they carry oxygen-based heterocyclic rings. While the nucleotides in the IMO consists of complex oligomers of pentoses linked to a heterocyclic base and a phosphate group, the *N*-glycans consist of units of hexoses linked by glycosidic bonds (Figure 6). Although *N*-glycans differ from oligonucleotide structures, X-ray crystal structure studies have shown that hydrogen bond interactions are important for the interaction between antagonists and the TLR-8 dimer (47). While CQN appears to bind to ssRNA40 and R848 ligands, the *N*-glycans seem to interact directly with the TLR-8 receptor.

The different ways by which CQN and *N*-glycans inhibit TLR-8 activation should also explain the difference in efficacy to attenuate TLR-8 induced activation of human MoDCs. CQN inhibits the production of IL-6, IL-10, and TNF-α in MoDCs. This strong impact of CQN probably is due to a complete binding and neutralization of ssRNA40. In contrast, the *N*-glycans only inhibited the secretion of IL-6 and did not reduce the secretion of IL-10 and TNF-α. Although the various *N*-glycans tested did not differently inhibit the ssRNA40 induced activation on TLR-8 expressing reporter cells, the partially demannosylated, and partially desialylated *N*-glycans had a stronger inhibitory effect on IL-6 secretion in MoDCs. This should be explained by the fact that MoDCs are carrying more pattern recognition receptors than TLR8. In previous studies it has been shown that the activation pattern of bLF on other TLRs is dependent on the sialylation and mannosylation composition of the *N*-glycans (20). This may explain the stronger impact of the partially demannosylated and partially desialylated *N*-glycans on IL-6 secretion in MoDCs which probably involve other receptors that sense glycans, such as C-type lectines (48).

This study was undertaken in our efforts to identify food components that can be used in anti-inflammatory diets. Targeting the inhibition of IL-6 by bLF has positive clinical implications, for instance for patients with rheumatoid arthritis that fail anti-TNF therapy (49). However, other diseases may benefit from this approach as well, such as Crohn’s disease, systemic lupus erythematosus, systemic sclerosis, and some cancers (50). The potential anti-inflammatory effects derived from a dietary component such as the *N*-glycans from bLF can have a great impact when used for therapeutic purposes. Compared to CQN, which can induce severe adverse reactions, such as retinopathy, psychiatric, cardiac, and neuromuscular adverse effects (30), bLF and its derivates, peptides and N-glycans, have no adverse effects (51, 52). Additionally, as shown in this study, *N*-glycans are selective inhibitors of TLR-8 activation whereas CQN is not and this treat is considered a challenge in the therapy of rheumatoid arthritis with antimalarials (53, 54). The prevention of adverse reactions while reducing or controlling inflammation is relevant for the long term use of a therapeutical treatment and the well-being of any patient.

## Conclusion

We show that *N*-glycans isolated from bLF are able to inhibit specifically TLR-8 and that this impacts the production of IL-6 in MoDCs. Our findings indicate that this impact is higher in MoDCs with partially demannosylated and partially desialylated *N*-glycans. Our findings demonstrate that the *N*-glycans are as effective as CQN in reducing production of IL-6. As TLR-8 over activation is involved in many inflammatory disorders, our data suggest that lactoferrin may fit into an anti-inflammatory diet.

## Data Availability Statement

All datasets generated for this study are included in the article/Supplementary Material.

## Ethics Statement

Blood sampling of human volunteers was conducted within the University Medical Center Groningen (UMCG), Netherlands. This research and consent procedure have been approved by the Ethical Review Board of the UMCG, as documented in the approved application “2007/255.” The patients/participants provided their written informed consent to participate in this study.

## Author Contributions

SF-L and RA performed the cell-based experiments. RV-W isolated and characterized the *N*-glycans. WA assisted with the MoDCs experiments. SF-L performed the data analysis. SF-L and PV outlined and wrote the manuscript. WA, SL, LD, and PV performed critical review of the manuscript. All authors have revised and approved the manuscript.

## Conflict of Interest

The authors declare that the research was conducted in the absence of any commercial or financial relationships that could be construed as a potential conflict of interest.

## Abbreviations

bLF: Bovine lactoferrin
Mo: Monocytes
MoDCs: Monocyte-derived dendritic cells
PBMCs: Peripheral blood mononuclear cells
TLR-8: Toll-like receptor 8.
