# Supplementary material for: Inhibitory Effects of Dietary N-Glycans From Bovine Lactoferrin on Toll-Like Receptor 8; Comparing Efficacy With Chloroquine
Source: Front Immunol. 2020 May 12;11:790. doi: 10.3389/fimmu.2020.00790 (PMC7235371; doi:10.3389/fimmu.2020.00790)

Supplementary Material

Figure S1. mRNA expression of TLR-8 in immature and mature MoDCs. The mRNA expression of TLR-8 in monocytes was compared with the TLR-8 mRNA expression in MoDCs. Results were normalized to GAPDH and are presented as mean of 5 independent experiments. Data is presented as median with interquartile range. Statistical differences were measured using Mann-Whitney test. Significant differences compared with the MoDCs expression were indicated with * $p < 0.05$.

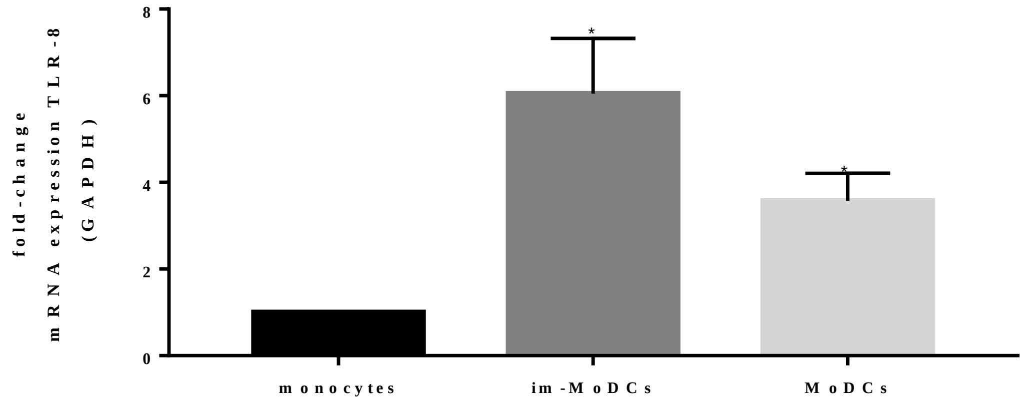

Figure S2. Dose response of ssRNA40 to the inhibitory effects of CQN and native *N*-glycans on the secretion of IL-6 in MoDCs. Cells were separately pre-incubated for 1 h with 2.5, 5 and 10 µg/mL of CQN and 6.25, 12.5 and 25 µg/mL of native *N*-glycans. Afterwards, cells were stimulated with 5, 10 and 15 µg/mL of ssRNA40. The secretion of IL-6 was measured by ELISA. Data is represented as mean ± SEM. Statistical differences were measured using One-way ANOVA and post hoc Tukey's test. Significant differences compared to ssRNA40 are indicated by **** $p < 0.0001$.

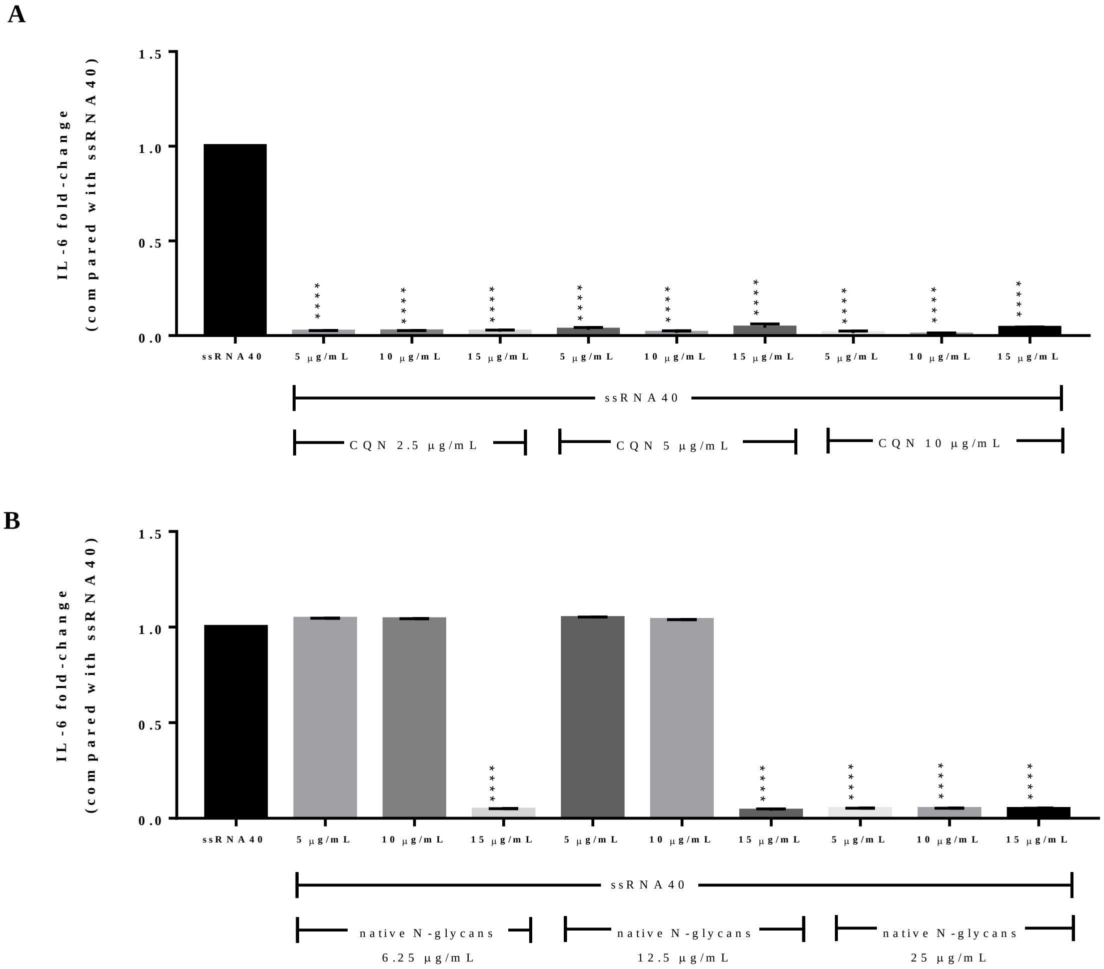

Supplement: Supplementary file 1 [file Data_Sheet_1.PDF]
